# Polyelectrolyte–Dye Interactions: An Overview

**DOI:** 10.3390/polym14030598

**Published:** 2022-02-02

**Authors:** Gulmi Chakraborty, Ajaya Bhattarai, Ranjit De

**Affiliations:** 1 Department of Chemistry, C. V. Raman Global University, Odisha 752054, India; gulmi.chakraborty@gmail.com; 2 Department of Chemistry, Mahendra Morang Adarsh Multiple Campus, Tribhuvan University, Biratnagar 56613, Nepal; 3 School of Material Science and Engineering, Gwangju Institute of Science and Technology, Gwangju 61005, Korea; 4 Department of Life Sciences, Pohang University of Science and Technology, Pohang 37673, Korea

**Keywords:** polyelectrolytes, dye, interaction parameters, polymer, polymer–dye interactions

## Abstract

Polyelectrolytes are polymers with repeating units of ionizable groups coupled with counterions. Recently, polyelectrolytes have drawn significant attention as highly promising macromolecular materials with potential for applications in almost every sector of our daily lives. Dyes are another class of chemical compounds that can interact with substrates and subsequently impart color through the selective absorption of electromagnetic radiation in the visible range. This overview begins with an introduction to polyelectrolytes and dyes with their respective definitions, classifications (based on origin, molecular architecture, etc.), and applications in diverse fields. Thereafter, it explores the different possible interactions between polyelectrolytes and dyes, which is the main focus of this study. The various mechanisms involved in dye–polyelectrolyte interactions and the factors that influence them are also surveyed. Finally, these discussions are summarized, and their future perspectives are presented.

## 1. Introduction

**Figure 1:**
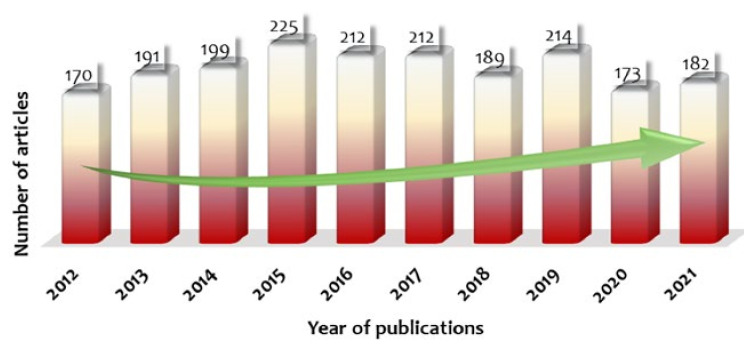
Number of publications per year in the area of polyelectrolyte–dye systems showing the consistency in scholarly interests. Data were obtained from ‘Scopus’ using the keywords ‘polyelectrolytes’, ‘dyes’, and ‘interactions’.

Dye-associated polyelectrolytes have found numerous applications from biology to textile industry. In the case where a dye is cytotoxic, it can be wrapped by polyelectrolytes to increase its biocompatibility and subsequently be delivered to biological cells [1]. Dyes entrapped within polyelectrolyte complexes are also used for pH sensing [2]. Dye–polymer interactions have revolutionized textile industry [3]. Edible dyes along with bio-polyelectrolytes have been widely used in food manufacturing [4]. The use of dyes along with proteins has also found widespread application in biological studies. Recently, dyes encapsulated within polymer nanoparticles have been used for inkjet printing [5]. Polyelectrolyte membranes have also been widely used for the separation of dyes from wastewater by manipulating polyelectrolyte–dye interactions [6]. In all these cases, the materials are designed by exploring the molecular interactions between dyes and polyelectrolytes. Thus, understanding these interactions is a prerequisite to design such materials with the desired properties. Several research groups have attempted to explore these interactions case by case. For example, the influence of the charge densities and molecular structures of dyes on the interactions between poly(dimethylaminoethyl methacrylate) and dis-azo dyes have been explored by Dragan et al. [7]. Mamchits et al. explored the role of polyelectrolytes in the aggregation behavior of cyanine dye [8]. Recently, Ketema et al. reviewed the intermolecular forces between dyes and polyesters [9]. However, these studies were specifically focused on certain dye–polyelectrolyte systems. Thus, a review that analyzes the interactions between dyes and polyelectrolytes and covers virtually all the possible dye–polyelectrolyte cases is required to provide a better understanding in this area. Furthermore, the consistency in the number of scholarly works published in the recent past, as presented in Figure 1, projects the ongoing progress and the future perspectives of this field. This observation also reveals that the progress is not precipitously growing. Hence, this overview, which summarizes the potentials of polyelectrolyte/dye systems in the light of molecular interactions, can be foreseen to enrich the information available and provide an easy-to-access resource.

### 1.1. Polyelectrolytes

Polyelectrolytes (PEs) form an interesting class of macromolecules that dissociate in polar solvents to produce a large number of charged groups and their corresponding counterions [10]. The smaller counter ions neutralize the repeating charged groups and preserve the electro-neutrality. In an uncharged state, the behavior of PEs resembles that of normal macromolecules; however, the dissociation of the ionic groups, even to a small extent, may lead to dramatic changes in their physico-chemical properties [11]. Thus, polyelectrolytes can exhibit both the properties of polymers and electrolytes, which is advantageous towards their interactions with various types of dye molecules. Such polymer behavior can be modulated by the partial or complete dissociation of the ionic groups, which subsequently alters the electrostatic interactions leading to deviations in their polymeric properties [12]. The physical properties of PEs, such as viscosity, diffusion coefficient, solubility, pH, ionization constant, and ionic strength, can be modified by the introduction of ionic moieties into the polyelectrolyte environment [13].

Due to their excellent water stability and ability to interact with oppositely charged macromolecules and surfaces, polyelectrolytes have been extensively used in various fields, from materials science and colloids to biophysics. Their predominant applications include usage in optoelectronic devices [14], solar cells [15], rheology modifiers [16,17], adsorbents [18], coatings [19,20], biomedical implants [21], colloidal stabilizers [22], suspending agents [23], and for drug delivery and pharmaceutical uses.

Polyelectrolytes can be classified into different categories depending upon their origin, charge, pH dependence, morphology, position of ionizable sites, and composition (Table 1). Some natural polyelectrolytes include carbohydrates, alginates, chitosan, carrageenan, pectin, and nucleic acids, while synthetic polyelectrolytes, such as poly(acrylic acid), poly(vinyl amine), poly(vinylsulfonic acid), and polyvinylpyridine, are also common.

Among the anionic polyelectrolytes, carboxylate –COO^−^, phosphonate ($-{\mathrm{PO}}_{3}H^{-},-{\mathrm{PO}}_{3}^{2-}$), and sulfonate ($-{\mathrm{SO}}_{3}^{-}$) are the most common functional groups, whereas cationic polyelectrolytes are mostly comprised of the primary, secondary, and quaternary amino (-NH_2_, =NH, and =$N^{+}$=) groups. The types of ionic groups, their counter ions, and the structures of the repeating units determine the properties of polyelectrolytes, such as their solubility in water and other polar and hydrogen-bonding liquids (alcohols, etc.), electrical conductivity, and rheology. Unlike nonionic polymers, these properties strongly depend on the pH, solvent permittivity, and ion content [24].

The electrostatic interactions (attraction/repulsion) between charges present on the monomeric units of polyelectrolytes lead these macromolecules to be rich in a variety of physicochemical properties. For instance, in the absence of added salts (ions), the electrostatic repulsion between the same charges of monomer units of a macromolecule can result in significant chain elongation, which can vary almost linearly with the degree of polymerization [25]. Due to the strong influence of the degree of polymerization on chain morphology transition (coiling to elongation), which results in an increase in chain size, the crossover to the semi-dilute polyelectrolyte solution regime can be achieved at much low polymer concentrations than in the case of nonionizable polymers [26].

### 1.2. Applications

As mentioned earlier, polyelectrolytes have found innumerable applications across various fields. These vast applications are concisely presented by considering the following generalized sectors.

#### 1.2.1. Polyelectrolytes for Optoelectronic Sensing Devices

*π*-conjugated polyelectrolytes, viz., polyaniline, polypyrrole, and polythiophene, with highly enhanced lifetimes and ultrafast cycle switching speeds (100 ms) were developed by Lu et al., and made electrochromic devices and actuators demonstrate drastically enhanced performance, cyclability, speed, and extended stability [14]. Large area light-emitting diodes (LEDs) developed with the conjugated polyelectrolyte poly(p-phenylene vinylene) were shown to exhibit electroluminescence in the green-yellow part of the visible spectrum [27]. The property of electroluminescence has enabled the use of polyelectrolytes in different LEDs. Poly(1,4-phenylene-ethinylenecarboxylate) complexed with dihexadecyldimethylammonium has been reported to exhibit blue luminescence [28]. Another blue light-emitting device based on a rigid-rod polyelectrolyte, namely, sulfonated poly(p-phenylene), which has both the luminescent and ionic properties, was reported with a low onset voltage (3.3 V) and external quantum efficacy up to 0.8% [29]. An LED based on poly(3-n-butyl-p-pyridylvinylene) and poly(p -phenylenevinylene) was found to largely increase the quantum efficacy of the device with a reduced operating voltage and prolonged lifetime [30]. A unique property of polyelectrolytes is their ability to self-assemble under a favorable ionic environment. Studies have found that the self-assembly of a negatively charged polyelectrolyte, poly(3-*α*-carboxylmethylthiophene), in the presence of a positively charged polyelectrolyte, poly(dihexyldipropargyl ammonium bromide), resulted in a multilayered heterostructure with enhanced photovoltaic effects [31].

#### 1.2.2. Polyelectrolytes in Multilayered Heterostructures

Multilayered polyelectrolyte films have been developed on surfaces by absorbing cationic and anionic polyelectrolytes alternatively, one layer at a time [32]. This technique, referred to as the layer-by-layer (LBL) technique, has led to the frequent usage of polyelectrolytes in several semiconductors and LEDs [33]. The fluorescence emission from polyelectrolytes can be effectively quenched by electron acceptors, leading to water soluble photo- and electroluminescent polyelectrolytes. For example, poly(2,5-bis(3-sulfonatopropoxy)-1,4-phenylene-alt-1,4-phenylene) with a poly(ethyleneimine) (PEI) polycation and poly(2,5-bis(2-(N,N,N-triethylammonium)-1-oxapropyl)-1,4-phenylenealt-2,5-thienylene) dibromide with poly(acrylic acid) (PAA), which both demonstrated enhanced fluorescence quenching as reported by Rubner and coworkers, were prepared via the LBL technique [34,35,36]. Based on the efficiency of polyelectrolytes in the development of multilayer heterostructures, these have been widely utilized to fabricate solar cells and photodetectors [15,37,38].

#### 1.2.3. Polyelectrolytes as Rheology Modifiers

Polyelectrolytes contain a large number of ions in their backbone, arousing electrostatic repulsion that can lead to two opposite consequences on their viscosity: (i) an increase in viscosity triggered by chain elongation and (ii) a decrease in viscosity caused by suppressed intermolecular interactions [39]. Upon the increase in the concentration of added salt (ions), the gradual transition from an elongated to a random coil conformation (decrease in viscosity) is favored due to the effective screening of the ionic charges of the monomeric units via counterion condensation. This also decreases the intermolecular electrostatic repulsion, i.e., the intermolecular attraction increases, which thereby raises their solution viscosity [40]. Polyelectrolytes have been hydrophobically modified to provide an improved thickening effect and for building intense three-dimensional networks [41]. Such modifications which involve the insertion of a few hydrophobic groups (<2 mol%) into the hydrophilic backbone, resulting in unique rheological properties compared to the unmodified counterparts [42,43]. The modified polyelectrolytes with tailored rheological properties have found various uses in the design of cosmetics, paints, and coatings [44]. Polyelectrolytes based on polyurathanes, hydroxyethylcellulose, and alkali swellable acrylates have demonstrated promising results in improving the performance and binding ability of water-borne coatings [45]. Polyacrylamides have also been hydrophobically modified to demonstrate a high viscosity yield under highly saline conditions, and have shown promising applications in oil field recovery [40,46].

### 1.3. Dyes

**Figure 2:**
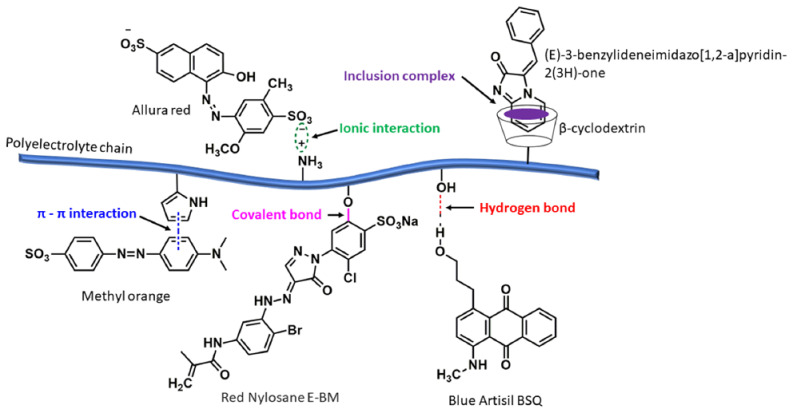
Schematic presentations of various molecular interactions between dyes and the functional/ionizable moieties of a polyelectrolyte chain.

The usage of color initiated at the dawn of time with the purpose of making art look beautiful; presently, it has achieved numerous applications. Dyes are the main constituents of color. Dyes are colored substances that can bind to the substrates to which they are applied. They are used to impart colors to fabrics, food stuffs, textiles, and other objects for their beautification and distinction [47]. They are often organic compounds and are soluble in water. Plants are usually the source of natural dyes, such as their roots, leaves, barks, fruits, wood, etc. Some common examples of natural dyes are jack fruits, turmeric, onion, henna (*Lawsonia inermis*), indigo, etc. Synthetic dyes can be derived from petroleum products, after cracking crude oil. Mauveine or aniline purple was the first synthetic dye, discovered by Perkin on 26 August 1856. The way that the colors of dyes correlate with their structures can be explained by different theories. The compounds containing chromophores are called chromogens. The intensity of color increases with the number of chromophores or the degree of conjugation [48]. The presence of certain functional groups facilitates the fixation of chromogens to the fabrics to be dyed. The groups that favor the permanent fixation of chromogens to the materials to be dyed are called auxochromes. Some examples of auxochromes include -OH, -NH_2_, -NR_2_, -SO_3_H, etc. Dyes are broadly used in the fields of industrial and personal care products, disease diagnosis, drug delivery, wastewater treatment, and pharmaceutical products, which are very useful in our daily life although some of them may also have side effects. Thus, it is very necessary to study these molecules and their interactions with other molecules, which may be electrostatic, hydrophilic/hydrophobic, H-bonding, covalent, non-bonding, etc. The nature and strength of these interactions are dependent on the properties of the dyes and their molecular structures. The interactions between surfactant-dyes or polyelectrolyte-dyes can be hydrophobic or hydrophilic in nature, where molecules pull due to low alliance with water; these interactions can also be electrostatic, where two particles attract or repel due to the presence of charges on the molecules [49,50]. The interaction of anionic dyes, e.g., tartrazine and cationic surfactants (dodecyltrimethylammonium bromide, cetyltrimrthylammonium bromide, etc.), were investigated using tensiometric techniques [51]. Hydrophobic interactions occur between nonpolar molecules, e.g., the interactions between hydrocarbon chains, and they cannot be solubilized in water. On the other hand, hydrophilic interactions occur between polar molecules, which can be solubilized in water or other polar solvents. Similarly, H-bonding occurs between two electronegative elements using an H-atom as a bridge. Schematic presentations of these various molecular interactions between dyes and polyelectrolytes are shown in Figure 2. Molecular interactions are the driving force behind the complexation between dye and polyelectrolytes, and the thermodynamic parameters related to this interaction can evaluate the stability of such metachromatic complexes [52,53] The mixing of polyelectrolytes with dyes can change the solution properties, such as surface tension, viscosity, conductivity, critical micellization concentration (*cmc*), the nature of spectra, phase behavior, and solution rheology [54]. A red shift of the monomer band of dye can be observed after interaction with cationic dyes. In a study, cationic dyes (rhodamine 6G, proflavine, acridine orange), upon interacting with anionic polyelectrolytes (sodium dextran sulfate, polyvinyl sulfate), formed a dye–polyelectrolyte complex that could be used for contaminant removal from sludges [55]. The intensity changes of fluorescent dyes, which can be recorded using any spectrofluorometer, are often monitored to determine the interactions between dye and polyelectrolytes [56].

#### 1.3.1. Classification of Dyes

As per the theoretical models and the knowledge on the electronic origins of color, dyes are categorized into four major classes based on their types of chromogens, namely, (a) donor-acceptor, (b) cyanine, (c) polyene, and (d) n → *π* transition [57]. There are various other ways that dyes are often classified [58]. Depending on their source of origin, dyes fall into two categories: natural or synthetic.

Based on their various industrial uses, dyes can be described as acid dyes, azoic dyes, basic dyes, direct dyes, dispersed dyes, reactive dyes, solvent dyes, sulfur dyes, vat dyes, and mordant dyes. A variety of dyes can be classified according to their chemical compositions or the nature of their nuclear structure, including acridine dyes, anthraquinone dyes, triarylmethane dyes, azo dyes, cyanine dyes, diazonium dyes, nitro dyes, nitroso dyes, phthalocyanine dyes, aniline dyes, eurhodin dyes, safranine dyes, xanthen.

**Figure 3:**
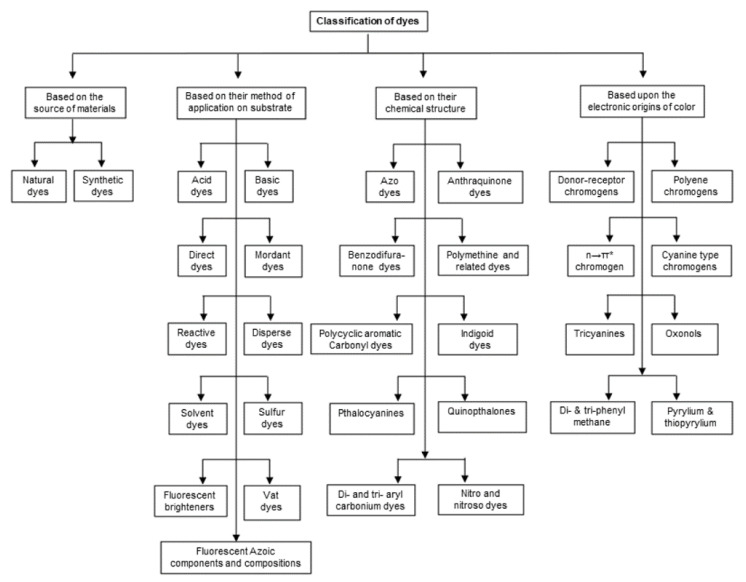
A summary of the classification of dyes based on different factors.

Depending on other miscellaneous factors, additional classifiable dyes are fluorescent dyes, oxidation dyes, fuel dyes, leather dyes, optical brighteners, leuco dyes, sublimation dyes, smoke dyes, inkjet dyes, and solvent dyes. A summary of the different classifications of dyes is presented in Figure 3.

#### 1.3.2. Applications of Dyes

Color originates from the spectrum of photons interacting with the spectral sensitivities of the light receptors in the eye. As mentioned earlier, dyes can be used in different sectors, including medicine, industrial areas, cosmetics, chemical analysis, dyestuffs, the food industry, textiles, dying, etc. Cyanine dyes are used as synthetic drugs in various ways, e.g., as cell growth inhibitors, photoreceptors, photorefractive materials, fluorescent sensors, etc. [59]. Reactive dying increases the fiber retention of dye. These dyes can also be used as fluorescent probes for living cells [60]. Depending upon the composition of the dye molecules, the coloring of hair can be temporary or permanent [61]. However, it should be noted that in all these applications, the interactions between dyes and substrates are inevitable. The following sections address these various interactions, restricted to polyelectrolyte–dye systems.

## 2. Polyelectrolyte–Dye Interactions

Polyelectrolytes and dyes comprise two of the most important classes of chemical compounds with the most versatile application in industrial chemistry. The interactions between polyelectrolytes and dye lead to formation of polyelectrolyte–dye complexes with modified physical and chemical properties. In the following sections, the applications of some materials prepared by the interactions between polyelectrolytes and cationic dyes, as well as polyelectrolytes and anionic dyes, are presented.

### 2.1. Polyelectrolyte–Dye Interactions and Their Applications

#### 2.1.1. Polyelectrolytes and Anionic Dyes

Dye removal remains one of the most challenging aspects of industrial waste management. The strong interactions between polyelectrolytes and dyes were pushed further by Cai et al. to develop a chitosan-based cationic polyelectrolyte microsphere (CCQM) for the ultra-efficient removal of Congo red (1500 mg g^−1^) and methyl orange (MO, 179.4 mg g^−1^) [62]. Based on the strong polyelectrolyte–dye interactions, hydrogels fabricated using poly([2-(acryloyloxy)ethyl] trimethylammonium chloride) and poly(ClAETA) with cellulose nanofibrillation (CNF) had an efficiency of 96% in the removal of methyl orange dye, which remains a major industrial contaminant [63]. Schwarze et al. developed polyelectrolytic emulsions based on quaternary ammonium surfactants and demonstrated a dye removal efficiency of 90% for methyl orange [64]. The dye–polyelectrolytic complex aggregates have an important role in determining the spectral behavior of the dye. It was observed that methyl orange demonstrated an absorption maximum at 368 nm in poly(l-ornithine) (PLO), compared to 462 nm in poly(vinyl benzyl triethylammonium chloride) (PVBTEA). This observation was attributed to the formation of larger aggregates in PLO compared to PVBTEA, which promoted electrolytic dye stacking via ion-pair formation [65].

Microgels are three-dimensional cross-linked structures of polymer colloidal particles with an adjustable size and strong response to environmental stimuli, such as pH, ionic strength, temperature, light, and ultrasound [66]. Self-assembled microgels comprised of poly(N-isopropylacrylamide-*co*-2-(dimethylamino) ethyl methacrylate) and sodium alginate (SA) have demonstrated a highly pH-sensitive response [66]. Methyl blue is an anionic hydrophilic dye molecule which has been reported to be adsorbed onto microgel core star ionic covalent organic polymers, polymers, fibrous materials, and cross-linked polymer particles with cavity and ammonium functionalization [62,67,68,69]. A quartz crystal microbalance (QCM) investigation of the interaction between anionic dyes and the SA/microgel multilayers in the aqueous phase revealed an enhanced electrostatic attraction between the dyes and the microgels deposited on the QCM sensor surface compared to that with SA in the multilayers, which caused the release of microgels from the self-assembled structure and a mass loss ratio of 27.6% [66]. This study showed a promising application of the QCM-based sensors in the detection of dye contaminants in wastewater. In another report, a linear polysaccharide chitosan (CTS) composed of *β*-(1-4)-linked D-glucosamine (deacetylated unit) and N-acetyl-D-glucosamine (acetylated unit), was chemically modified to form a cationic polyelectrolyte, viz., N-[(2-hydroxy-3-trimethylammonium)propyl]chitosan chloride (HTCC) [70]. A comparative investigation of the interaction of three anionic dyes, viz., Reactive Black 5, Reactive Blue 19, and Reactive Red 195, with HTCC and organoclay-modified montmorillonite (OMMT) demonstrated a high efficiency of dye exclusion (>91%) compared to sole polyelectrolyte and organoclay adsorbents. The study further showed that structurally distinct anionic dyes localized at separate sites within the hybrid organoclay adsorbents, enabling the simultaneous adsorption of different dyes with improved efficiency [70]. Thus, the materials designed via the interactions between polyelectrolytes and cationic dyes have exhibited success in various fields.

#### 2.1.2. Polyelectrolytes and Cationic Dyes

Cationic dyes dissociate into positively charged ions and negative counterions in aqueous solutions and have been extensively explored to study their interaction with anionic polyelectrolytes. The strength of this interaction can be measured by the magnitude of metachromasy induced in its spectroscopic profile. Metachromasy, or the blue shift in the absorption spectrum, is one of the most common methods for spectroscopic detection of polyelectrolyte–dye interactions. Higher metachromic effects imply a stronger degree of interaction. Toluidine blue (7-amino-8-methylphenothiazin-3-ylidene)-dimethylammonium chloride) and methylene blue (3,7-bis(dimethylamino)-phenothiazin-5-ium chloride) both form a strong 2:1 dye–polyelectrolyte complex with polyacrylic acid polymer (PAA), exhibiting large hypsochromic shifts of 57 nm and 67 nm, respectively, in their UV-vis profiles. Consequently, due to the more hydrophobic nature of methylene blue, it formed a more stable complex with PAA compared to toluidine blue: the stability constants were 5332 dm^−3^/mol and 4358 dm^−3^/mol for methylene blue and toluidine blue complexes, respectively, at 298 K [71]. The interaction of toluidine blue with poly(potassium vinyl sulphate) (PPVS) resulted in observed metachromacy at 105 nm with a distinct color change of the blue uncomplexed form with a maximum absorption at 635 nm to a red-violet toluidine blue–PPVS complex with maximum absorption at 530 nm [72]. The metachromatic action of cationic dyes is particularly helpful in determining the charges of biopolymers and proteins [73]. The specific interaction of the polymerized cationic dye azure A and the biological polyelectrolyte DNA was utilized to detect and discriminate DNA damage [74]. The electrostatic forces and the difference in the negative charges on the repetitive polysaccharide units of sodium heparin and sodium alginate led to different degrees of metachromasy induced in the cationic dyes azure B and toluidine blue [75,76]. While azure B bonded more strongly with sodium alginate, a more favorable interaction was observed between toluidine blue and sodium heparin. The interactions between poly(2-acrylamide-2-methyl-1-propanesulfonic acid) (PAMPS) and poly(diallyl dimethyl ammonium) chloride (PDDA) and the highly versatile cationic dyes methylene blue (MB) and methyl orange (MO) have been employed for the purification of colored wastewater by the polymer-enhanced ultrafiltration (PEUF) technique [6]. The maximum removal efficiency under optimal conditions (pH 6.0, initial MB and MO concentrations of 3.5 mg L^−1^ and 80 mg L^−1^, respectively) was reported to be 98% and 90% for MB and MO, respectively, together with an ultrafiltration membrane (molecular weight cut off value: 10 kDa). Polystyrene sulfonate (PSS) adsorbed on laterite soil (polymer modified laterite, PML) showed efficiency in the removal of methyl blue (83%) and crystal violet (92%) [77].

Dye removal remains one of the most challenging aspects of industrial waste management. The strong interactions between polyelectrolytes and dyes were pushed further by Cai et al. to develop a chitosan-based cationic polyelectrolyte microscope (CCQM) for the ultra-efficient removal of Congo red (1500 mg g^−1^) and methyl orange (MO, 179.4 mg g^−1^) [78].

## 3. The Two Basic Mechanisms of Polyelectrolyte–Dye Interactions

**Figure 4:**
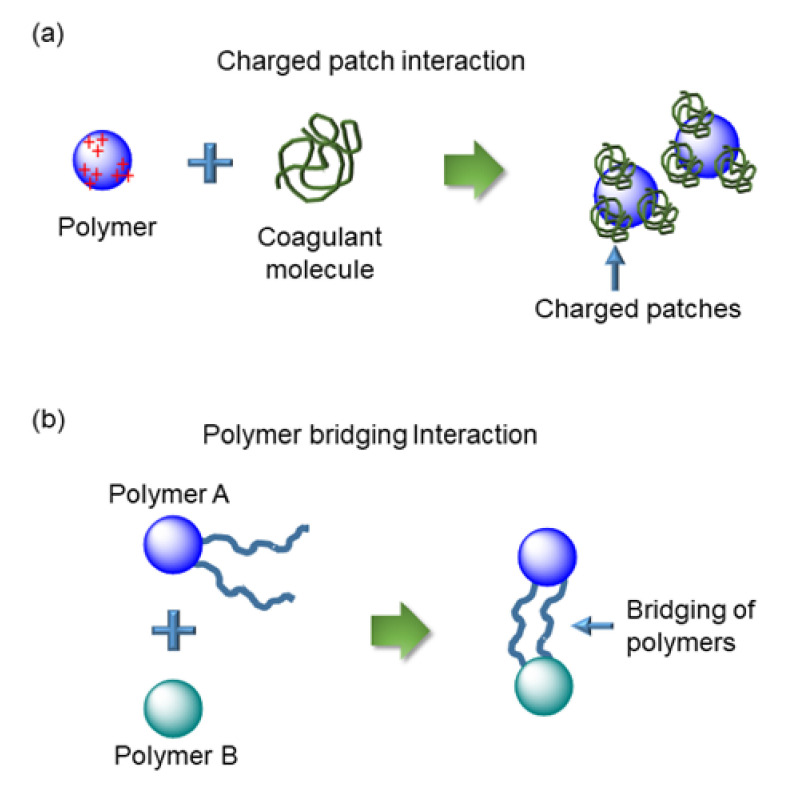
Schematic representation of (**a**) the charged patch interaction, where oppositely charged coagulant molecules bind electrostatically to polyelectrolytes at specific charged patches, and (**b**) the polymer bridging interaction, where the polymeric chain from one polyelectrolyte is extended to adsorb onto another polymer, resulting in a bridging interaction.

Interestingly, the myriad of polyelectrolyte–dye interactions has been found to follow either of two basic mechanisms of interaction: the charged patch interaction (Figure 4a) and the polymer bridging interaction (Figure 4b) [79,80].

### 3.1. Charged Patch Interaction

This mechanism broadly refers to the formation of a ‘charged patch’ due to the electrostatic interaction between relatively low molecular weight polyelectrolytes adsorbed on oppositely charged surfaces (Figure 4a). The patch is electrostatically attracted to the bare regions of other oppositely charged particles (coagulant molecules), which favors flocculation. The flocculation of polyelectrolytes is aided by their adsorption onto the porous surfaces, such as organoclay [70]. The interaction of dye molecules with the adsorbed polyelectrolyte has been found to be more efficient than with pure electrolytes. Depending on the interaction mechanisms, the polymeric adsorption may be physical or chemical. While physical absorption involves relatively weak bonds, e.g., van der Waal interactions, chemisorption includes stronger covalent bonding between the polyelectrolyte and substrate [81].

### 3.2. Polymer Bridging

Polymer bridging refers to the mechanism whereby a polyelectrolyte is adsorbed simultaneously on more than one polymeric surface (Figure 4b) [82]. High molecular weight polyelectrolytes with linear chains commonly based on polyacrylamide are reported to be ideal candidates for this kind of interaction [83]. However, the high charge density associated with high molecular weight polyelectrolytes has a contrasting effect on flocculation, due to the electrostatic repulsion between like charges. Still, such materials are used for interaction with dye molecules to remove them during wastewater treatment [3,84].

## 4. Parameters That Influence Polyelectrolyte–Dye Interactions

### 4.1. Polyelectrolyte Concentration

The stoichiometric ratio of the polycations and polyanions of polyelectrolytes plays an important role in maneuvering the mechanistic pathways of their interaction with dyes [85]. Azure B (AB) formed a 1:1 complex with sodium heparin, i.e., the binding of the dye cation at all potential anionic sites led to the formation of a ‘card-pack’ stacking of the dye monomers on the surface of the polyelectrolyte. However, in the case of NaAlg, binding at alternate site resulted in 2:1 stoichiometry [75]. A similar 1:1 complex of *N*,*N*ˊ- diethylpseudoisocyanine chloride (PIC) with polymethacrylate, poly(styrenesu1fonate), and DNA (native and denatured) did not exhibit any blue shift or metachromasia in the absorption spectra, while polyacrylate and poly(viny1sulfate) formed compounds with polyanion/dye stoichiometry of 2:1, forming staggered aggregates which exhibited a sharp and red-shifted J-band in the UV-vis spectra [86]. On the other hand, the incubation of carboxyfluoroscein (CF) on a model with 24 hyaluronan/polylysine (HA/PLL) multilayers resulted in 13 mM of CF loaded in the multilayer, compared to 0.5 mM in tris(hydroxymethyl)aminomethane (TRIS) buffer [87]. Emission studies revealed that the interaction of the CF molecules with the free amino groups of PLL along with the CF –CF self-interaction contributed to the cooperative binding and polyadsorption of the dye molecule. With an increased dye concentration at low PAA concentrations (up to 0.4 mM), the attachment of dye molecules remained unfavorable. Whereas, for the same PAA concentrations (up to 0.4 mM), CV was favorably adsorbed onto the monomer units of PAA. Increasing the polymer concentration beyond the threshold resulted in the sharp attachment of the dye molecules to the polymer [88]. The dye safranin T (ST) was found to bind to the polymer PANH4 to a greater extent than eriochrome blue black dye (EBBT). ST is less bulky, which favors its preferential attachment to the polymer and efficiency compared EBBR when the feed concentrations of both the dyes were the same.

### 4.2. Dye Concentration

A spectroscopic study on the electrostatic interactions (binding) of the cationic dyes rhodamine 6G (R6G), acridine orange (AO), bisindolenylpentamethine (Cy5), and 1,1′-diethyl-2,2′-cyanine (PIC) to the anionic polyelectrolyte polystyrene sulfonate (PSS) showed that Cy5 bonded to PSS with a 10 nm hypochromic shift in the absorption spectrum, provided the dye/polyelectrolyte ratio was less than 0.1 [89]. The charges on PSS facilitate the polarization of the *π* electronic charges, resulting in dye–electrolyte bonding. A higher dye/polyelectrolyte ratio resulted in the formation of H aggregates and, consequently, the rejection of the dye. On the other hand, the adsorption of the non-fluorescent dye 1,1′-diethyl-2,2′-cyanine (PIC) on polystyrene sulfonate (PSS) resulted in weak fluorescence emission due to the formation of J-aggregates of the PIC complex at the optimal dye/polymer ratio of 0.55. An increased concentration of dye resulted in the destruction of the J-aggregates [89].

### 4.3. pH of Reaction Media

pH is one of the most important factors governing the binding of dyes with polyelectrolytes. In a study of poly(acrylic acid) (PAA) with the cationic dyes methylene blue (MB) and toluidine blue (TB), it was found that the spectrum of methylene blue was indifferent to pH below the pKa of the polyelectrolyte (4.65). The reason for this is the predominant acidic nature of poly(acrylic acid) at a low pH (2–3.65), which forbids preferential binding with the cationic dye due to electrostatic repulsion [71]. At a pH above 4.42 (pH > pKa), the monomeric absorption band of the methylene blue decreased with the corresponding growth of a metachromatic band showing the binding of the cationic dye molecules with the anionic polyelectrolyte. Interestingly, at a high pH (>10.86), the monomeric band absorption reappeared. The authors concluded that the dye–polyelectrolyte binding was weakened at a higher pH due to the complete binding of the dye to the oppositely charged polyelectrolytic surface, resulting in free monomers. A similar trend in the absorption profile of methylene blue was observed with polyacrylamide and sodium polyacrylate [90]. Higher pH may lead to the hydrolysis of cationic polyelectrolytes, lowering the charge density and thereby weakening the dye–polyelectrolyte interaction [72]. Studies on crystal violet and the polyelectrolyte poly(acrylic acid) (PAA) show that crystal violet (CV) completely binds with PAA above pH 3. The dissociation of the dye and the –COOH groups of PAA is prevented in highly acidic conditions (pH < 3), leading to lower dye retention [88]. While non-electrostatic effects dominate the binding of dyes to PANH4, at a higher pH or in an alkaline medium, the same is triggered by electrostatic effects due to the higher dissociation of PANH4. The decrease in the binding of the EBBR dye in an alkaline medium is propelled by the repulsion between hydroxide and the carboxylic groups. However, the attachment of safranin T (ST) is favored at a pH beyond 5. This observation may be attributed to the competition between H^+^ and ST to be affixed to the polymer, which arises due to their protonation, thereby favoring the dye attachment [88].

### 4.4. Influence of Electrolytes

**Figure 5:**
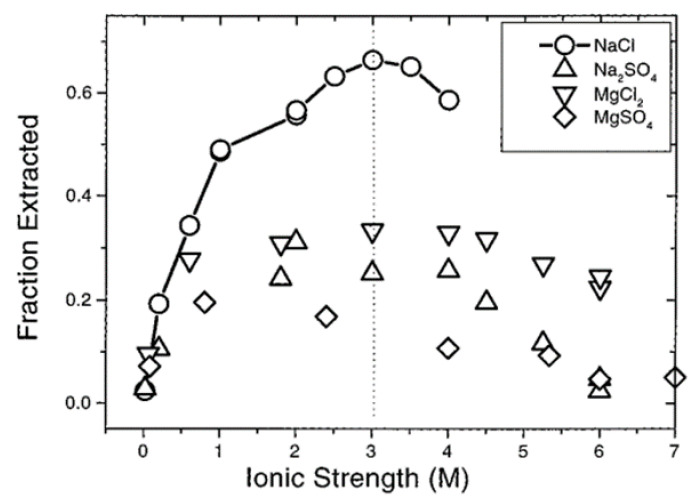
Fraction of dye removed from a surface dipped in 0.1 M poly(ethylenimine) for 30 min, submerged in 0.2 M poly(styrenesulfonate) for 20 minutes, immersed in 1 mM Ingrain Blue 1 (dye) for 20 min, then re-immersed in the original 0.2 M poly(styrenesulfonate) solution for 20 min versus the ionic strength of MgCl_2_(∇), Na_2_SO_4_(∆), and MgSO_4_(◊). Reproduced with permission from [92]. Copyright 1998 American Chemical Society.

The introduction of electrolytes or, in other words, the variation of ionic strength brings about a profound conformational change in the dye–polyelectrolyte interaction. A fluorescence investigation of the interaction between sodium copoly(ethy1 acrylate-acrylic acid) and the fluorescent dye 6-p-toluidinonaphthalene-2-sulfonate (TNS) showed that in the absence of salt/electrolytes, no emission spectra was observed, indicating that the TNS dye was completely quenched. The authors remarked that the polyelectrolyte assumed a linear conformation, and the hydrophobic interaction between the polyelectrolyte and the TNS molecules was overridden by the strong electrostatic repulsion exerted by the sulphonate head groups of the TNS molecule and the negatively charged carboxylic sites on the polyelectrolyte. The addition of salt caused the shielding of the counterion around the polyelectrolyte, which screened the aforementioned electrostatic repulsion between the dye and polyelectrolyte molecules, leading to a profound interaction and consequent increase in emission maxima [91]. Nandini et al. observed a similar phenomenon while studying the effect of salt concentration on the binding of methyl orange with cationic polyelectrolytes [90]. The complex interactions between dyes and polyelectrolytes (polyions) in multilayers are more prominent in polymer solutions over the polymer adsorption process, resulting in efficient dye extractions (Figure 5). A comparative study of the effect of electrolyte (NaCl, Na_2_SO_4_, MgSO_4_, and MgCl_2_) concentrations on the dye loading capacity of a poly(styrenesulfonate) multilayer revealed that an intermediate salt concentration facilitated the significant removal (as high as 60% NaCl) of the dye from the polyelectrolyte surface, with maximum extraction occurring at Debye length 2 Å [82]. At lower or higher salt concentrations, the dye loading was found to be relatively less. MgCl_2_, Na_2_SO_4_, and MgSO_4_ showed approximately 30%, 25%, and 20% dye extraction, respectively. At low salt concentrations, an increase in the persistent length of the polyelectrolyte led to a lesser extent of interaction between the charged dye molecule and the polyions, while at higher salt concentrations, the electrostatic interactions were diminished. The absorbance profile of poly(styrenesulfonate) showed a slight increase in the 1:1 and 2:2 salts, while a decreasing trend was reported with an increased 2:1 salt concentration. This intriguing result was attributed to the ‘charge reversal’ on the polymer or surface facilitated by the ions from salt [92].

### 4.5. The Role of Surfactants

The addition of surfactants in dye–polyelectrolyte systems can lead to competitive binding of the surfactant with the polyelectrolyte, thereby releasing the dye molecule. This technique is used extensively to recover dye from wastewater [93]. The polyelectrolyte– surfactant interaction is governed by the flexibility of the polyelectrolyte, its charge density, the extent of hydrophobicity imparted by the nonpolar part of the polyelectrolyte, and the surface area of the polar head groups of the polyelectrolyte [94]. Increasing the concentration of the surfactant sodium lauryl sulphate (SLS) caused the reversal of metachromacy or a red shift in the absorption maxima in methyl orange–polyelectrolyte mixtures using poly(*N*-methy-4-vinylpyridinium iodide) (PM4VPI), poly(vinylbenzyltriphenyl phosphonium chloride) (PVBTPPC) and poly(*N*-methy-4- vinylpyridinium iodide) (PM2VPI), indicating the release of MO with absorption of monomeric dye reappearing at an SLS concentration of 10^−4^ M [90]. A similar action of the surfactants SDS and sodium dodecylbenzenesulfonate (SDBS) was also observed in a complex of the anionic polyelectrolyte sodium heparinate (NaHep) and the cationic dye azure AB [76]. Alternatively, a methyl orange–sulfonated polystyrene (SPS) interaction was used as a spectroscopic probe to monitor the competitive binding and stability of the cetyltrimethylammonium bromide (CTAB)–SPS complex [95]. In another report, a complex of the cationic polyelectrolyte poly(3-(4-methyl-30-thienyloxy) propyltrimethylammonium and the anionic dye 8-hydroxy-1,3,6-pyrenetrisulfonic acid trisodium salt (HPTS) was utilised to sense the anionic surfactants sodium dodecylbenzenesulfonate (SDBS) and sodium dodecyl sulphate (SDS) via colorimetry and fuorescence spectroscopy [96].

The polyelectrolyte–dye interaction in the presence of surfactants is particularly important in the extraction of water-insoluble dyes, which are one of the major contributors to water pollution. The solubilization of the water-insoluble dyes *o*-(2-amino-1-naphthylazo)toluene (OY) and 1-pyrenecarbaldehyde (PyA) in mixed solutions of an anionic polyelectrolyte, viz., poly(styrenesulfonic acid), and cationic surfactants, viz., dodecyltrimethylammonium bromide (DTAB) and tetradecyltrimethylammonium bromide (TTAB), were investigated in [97]. Sodium dextran sulfate (DxS) exhibited pronounced binding of the surfactant ions, while the attachment to poly(styrenesulfonic acid) was diminished. The DxS/surfactant complexes exhibited a higher solubilization capacity per bound surfactant ion than the polystyrene/surfactant complexes for both *o*-(2-amino-1-naphthylazo) toluene (OY) and 1-pyrenecarbaldehyde (PyA) [97].

A polyelectrolyte enhanced ultrafiltration (PEUF) investigation on the retention of methyl orange in the presence of cetyltrimethyl ammonium chloride (CTAB) monomers showed a high degree of dye rejection by the polyelectrolyte polyethylene glycol (PEG) [98]. The enhanced rejection experienced by the MO dye was reported to be due to the development of H aggregates in the aqueous media.

## 5. Conclusions and Future Perspective

Polyelectrolytes are composed of a *π*-conjugated backbone with repeating ionic units, at the periphery or on the body, attached to counterions. The dissociation of the ionic units in aqueous solution gives rise to ionic conductance and solubility, which shows promise in highly versatile applications. The tunable features of polyelectrolytes, such as absorption, photoluminescence, and semi-conductor properties, have led to their multi-dimensional applications, from biosensors to optoelectronic devices. The interaction with dye is particularly interesting in view of the dye extraction process, which is one of the major challenges imposed by the textile industry. Each year, non-biodegradable dyes are released in enormous quantities by the textile industries as industrial effluent into water bodies, threatening the aquatic fauna. The polyelectrolyte–dye interaction has shown considerable promise in the dye extraction process; hence, it opens a new pathway to address this major pollution threat. This review provided an overview of the polyelectrolyte–dye interaction and its major applications. More research is warranted in this field for the optimal use of the potential of polyelectrolytes. The interaction of synthetic polyelectrolytes with different dyes should be further studied for developing designer complexes with applications in the commercial drug development field and optoelectronics.

## Figures and Tables

**Table 1:** Classification of polyelectrolytes based on different criterion.

Criterion	Classification		Examples
Origin	Natural		Protein
	Semi-synthetic		Xanthan Gum
	Synthetic		Poly(styrene sulfonic) acid
Charge	Polycation		N-[(2-hydroxy-3-trimethylammonium)propyl] chitosan chloride (HTCC)
	Polyanion		Poly(sodium styrene sulfonate)
	Polyampholyte		Protein
pH dependence	Strong: pH-independent charge		Poly(vinyl sulfate)
	Weak: pH-dependent charge		Poly(ethyleneimine)
Morphology	Rigid rod		Poly(2,2’-disulfonyl-4,4’-benzidine terephthalamide) (PBDT)
	Spherical		Globular proteins
Position of ion sites	Linear	Integral (Ions on the backbone)	Poly(2,20-disulfonyl-4,40-benzidine terephthalamide
		Pendant (Ions at the periphery or sidechain)	Poly(2-methacryloyloxyethyl 4-vinyl pyridinium bromide)
	Branched/crosslinked		Poly(4-styrenesulfonic acid-*co*-maleic acid) (PSS-*co*-MA) *co* polyethylene glycol (PEG)
Composition	Homopolymer		
	Copolymer		

## Data Availability

The data presented in this study are available on request from the corresponding author.
